# Plasma irisin is elevated in type 2 diabetes and is associated with increased E-selectin levels

**DOI:** 10.1186/s12933-017-0627-2

**Published:** 2017-11-09

**Authors:** Karan S. Rana, Chathyan Pararasa, Islam Afzal, David A. Nagel, Eric J. Hill, Clifford J. Bailey, Helen R. Griffiths, Ioannis Kyrou, Harpal S. Randeva, Srikanth Bellary, James E. Brown

**Affiliations:** 1 Aston Research Centre for Healthy Ageing and School of Life and Health Sciences, Aston University, Birmingham, B4 7ET UK; 2 Aston Medical Research Institute, Aston Medical School, Aston University, Birmingham, B4 7ET UK; 3 Warwickshire Institute for the Study of Diabetes, Endocrinology and Metabolism (WISDEM), University Hospitals Coventry and Warwickshire NHS Trust, Coventry, CV2 2DX UK; 4 Translational & Experimental Medicine, Division of Biomedical Sciences, Warwick Medical School, University of Warwick, Coventry, CV4 7AL UK; 5 Department of Diabetes and Endocrinology, Diabetes Outpatient Clinics at the Heart of England NHS Foundation Trust, Birmingham, B9 5SS UK; 6 Faculty of Health and Medical Sciences, University of Surrey, Guildford, GU2 7XH UK

**Keywords:** Irisin, Type 2 diabetes, Soluble E-selectin, Endothelial

## Abstract

**Background:**

Irisin is a hormone released mainly from skeletal muscle after exercise which increases adipose tissue energy expenditure. Adipocytes can also release irisin after exercise, acting as a local adipokine to induce white adipose tissue to take on a brown adipose tissue-like phenotype, suggesting that irisin and its receptor may represent a novel molecular target for the treatment of obesity and obesity-related diabetes. Previous reports provide conflicting evidence regarding circulating irisin levels in patients with type 2 diabetes (T2DM).

**Methods:**

This study investigated plasma irisin concentrations in 79 T2DM individuals, assessing potential associations with measures of segmental body composition, markers of endothelial dysfunction and peripheral blood mononuclear cell telomere length (TL).

**Results:**

Resting, overnight-fasted plasma irisin levels were significantly higher in this group of T2DM patients compared with levels we previously reported in healthy volunteers (p < 0.001). Moreover, plasma irisin displayed a positive correlation with body mass index (p = 0.04), body fat percentage (p = 0.03), HbA1c (p = 0.03) and soluble E-selectin (p < 0.001). A significant negative association was observed between plasma irisin and visceral adiposity (p = 0.006) in T2DM patients. Multiple regression analysis revealed that circulating soluble E-selectin levels could be predicted by plasma irisin (p = 0.004). Additionally, cultured human umbilical vein endothelial cells (HUVEC) exposed to 200 ng/ml irisin for 4 h showed a significant fourfold increase in E-selectin and 2.5-fold increase in ICAM-1 gene expression (p = 0.001 and p = 0.015 respectively), and there was a 1.8-fold increase in soluble E-selectin in conditioned media (p < 0.05).

**Conclusion:**

These data suggest that elevated plasma irisin in T2DM is associated with indices of adiposity, and that irisin may be involved in pro-atherogenic endothelial disturbances that accompany obesity and T2DM. Accordingly, irisin may constitute a potentially novel therapeutic opportunity in the field of obesity and cardiovascular diabetology.

## Introduction

Irisin is a recently identified hormone derived from the fibronectin type III domain-containing (FNDC5) gene that is released mainly from skeletal muscle after exercise or exposure to cold [1–3]. The ability of irisin, via yet undetermined receptors, to reprogram white adipose tissue (WAT) cells to take on the phenotype of brown adipose tissue (BAT) has been proposed as a potential therapeutic target for metabolic disorders, including obesity and type 2 diabetes (T2DM) [4–7]. Indeed, exposure to irisin leads to suppressed expression of several genes characteristic to WAT, whilst there is a concomitant increase in uncoupled respiration via induction of uncoupling protein-1 (UCP1) expression and altered expression of other established BAT genes [8–10]. Thus irisin can promote energy expenditure via thermogenesis [11]. Release of irisin has also been described from adipose tissue, suggesting that this hormone is an adipokine, as well as a myokine [12, 13]. Irisin release from WAT, as from muscle, is stimulated by exercise and reduced in fasted animals [12]. To date, there is conflicting evidence regarding possible association(s) between circulating irisin and body mass index (BMI): some data indicate a positive correlation [14], whilst other studies have found either no association [15] or a negative association [16]. Moreover, the role of irisin in T2DM remains unclear. Initial studies reported decreased circulating irisin levels in T2DM patients compared to healthy individuals [17, 18], whereas studies performed in obese individuals (some of whom had T2DM) have reported elevated levels [14, 19]. Recent data indicate that irisin may be involved in cardiovascular physiology, with evidence emerging that it plays a role in atherosclerosis [20–22] and can predict cardiovascular disease (CVD) risk [23, 24] This highlights the potential significance of irisin as a biomarker in T2DM patients who are at an increased CVD risk [25]. Moreover, we previously reported that plasma irisin levels can predict TL, a marker of biological ageing in healthy non-obese individuals, suggesting that irisin may provide a marker of ageing, as well as energy balance [26]. Since irisin is emerging as a new factor implicated in the pathophysiology of obesity and a potential opportunity to treat obesity, further appreciation of the role of irisin in obesity-related diabetes is required, particularly in relation to factors implicated in endothelial dysfunction and atherosclerosis, such as E-selectin.

E-selectin is a key adhesion molecule which alongside other endothelial adhesion molecules, serves to anchor leukocytes to the endothelium in inflammation and elevated soluble E-selectin levels have been identified in hypertension, diabetes and hyperlipidemia [27]. E-selectin is not constitutively expressed by endothelial cells [28], but is known to be expressed and released from activated endothelium both in vitro [29] and in vivo [30]. E-selectin expression is stimulated by exposure to inflammatory molecules including tumour necrosis factor-alpha (TNF-α) and interleukin-1 (IL-1) [31].

The present study investigated plasma irisin levels in a cohort of T2DM patients, examining associations between plasma irisin, indices of adiposity, ageing and circulating markers of endothelial dysfunction. The effects of exposure of primary cultured human endothelial cells to high irisin levels on expression and secretion of soluble forms of adhesion molecules was also investigated.

## Materials and methods

### Study participants

A group of 79 individuals with T2DM (42 males, 37 females; mean BMI 31.5 ± 5.4 kg/m^2^) was recruited from the diabetes outpatient clinics at the Heart of England NHS Foundation Trust, Birmingham, UK. The study exclusion criteria included pregnancy, recent hospitalization, other significant disease (e.g. cancer or immune disorders), and other significant treatments (e.g. oral corticosteroids). All subjects were asked to fast overnight for a minimum of 8 h and to refrain from exercise for at least 12 h prior to study sampling. The study was approved by the Aston University Research Ethics Committee and the Staffordshire NHS Research Ethics Committee and written informed consent was given by all participants according to the principles of the Declaration of Helsinki.

### Anthropometric and biochemical measures

Segmental body composition was measured using bioelectrical impedance analysis (BIA) (BC-601 Bioimpedance Analyser Tanita^®^). Analyses included segmental fat mass (FM), fat free mass (FFM) and a calculated visceral fat score (calculated by the manufacturer’s software; a score of 1–12 is considered healthy; 13–59 indicates excess visceral fat) in all subjects. Abdominal fat and fat free muscle readings are subtracted from other segmental readings to estimate the body trunk. Body weight was measured to the nearest 0.1 kg and height to the nearest 1 cm, whilst BMI was determined as body weight in kilograms divided by the square of the height in metres (kg/m^2^). A fasting sample of venous whole blood was collected into K^+^-EDTA coated tubes (Vacutainer, Becton–Dickinson, UK). Plasma was separated by cooled centrifugation (1300*g* for 10 min) and samples were stored at − 80 °C until analysed. For telomere length analysis, genomic DNA extraction was performed on whole blood aliquots using the QIAamp^®^ DNA blood mini kit (Qiagen, UK). DNA was resuspended in elution buffer (10 mM Tris·Cl; 0.5 mM EDTA; pH 9.0). Isolated DNA was quantified using the NanoDrop-1000 (NanoDrop Technologies, USA) and diluted in pure water to a concentration of 5 ng/µl and stored at − 80 °C.

Fasting whole blood glucose was measured using an Accucheck Advantage blood glucose meter. Glycated haemoglobin (HbA1c) was measured by mass spectrometry. Plasma irisin (Phoenix Peptides, Germany), leptin, soluble E-selectin, soluble thrombomodulin, C-reactive protein (CRP) (R&D Systems, UK) and insulin (Mercodia, Sweden) concentrations were all assessed by ELISA following protocols provided by the manufacturers. The homeostatic model assessment (HOMA) method was used to assess β-cell function (HOMA-β), and insulin resistance (HOMA-IR) was used to derive insulin sensitivity (HOMA-S) as previously described [32], according to the following equations:

$${\text{HOMA{-}}}\upbeta \% = {{\left[ {20 \times {\text{ fasting}}\;{\text{insulin }}\left( {{\text{mIU}}/{\text{l}}} \right)} \right]}/{{\text{Glucose}}\left( {{\text{mmol}}/{\text{l}}} \right) - 3.5}}$$

$${\text{HOMA{-}IR }} = \left[ {{\text{Glucose }}\left( {{\text{mmol}}/{\text{l}}} \right) \, \times {\text{ fasting insulin }}\left( {{\text{mIU}}/{\text{l}}} \right)} \right] / 2 2. 5$$

$${\text{HOMA{-}S}} = { 1}/{\text{HOMA{-}IR}}$$

Relative telomere length analysis was measured using an established real-time polymerase chain reaction (RT-PCR) to produce a relative expression ratio of telomeric DNA to genomic DNA control (T/S ratio) [26, 33].

### Cell culture and gene expression analysis

Early passage primary human umbilical vein endothelial cells (HUVECs) (Caltag Medsystems, UK) were grown in supplemented proprietary human large vessel endothelial cell basal medium (Caltag Medsystems, UK) in a 37 °C, 5% CO_2_ humidified incubator. Cells were seeded at 1 × 10^5^ cells per well in a 12-well plate and allowed to attach for 48 h before exposure to 0, 20 and 200 ng/ml recombinant human irisin (Caymen Chemicals, USA) for 4 or 24 h. Total RNA was isolated using EZNA RNA Isolation kit (VWR, UK), and treated with DNase (Promega, UK) to remove any traces of genomic material. RNA quantification was performed using the Nanodrop 1000 (Thermofisher). Samples (500 ng) of total RNA were reverse transcribed using Precision nanoscript™ reverse transcriptase (Primerdesign, Southampton UK) and oligo _d_T primers (PrimerDesign, Southampton, UK). cDNAs were amplified using a Stratagene MX3000P thermal cycler in a standard 40-cycle SYBR^®^ green real-time PCR reaction followed by a melt curve analysis to assess amplicon specificity. Gene expression was assessed with the following primers, E-selectin (sense AGAGGTTCCTTCCTGCCAAG, antisense CAGAGCCATTGAGGGTCCAT), P-selectin (sense CGCCTGCCTCCAGACCATCTTC, antisense CTATTCACATTCCAGAAACTCACCACAGC), ICAM1 (sense GACTCCAATGTGCCAGGCTT, antisense TAGGTGCCCTCAAGATCTCG) and PECAM1 (sense ATTGCAGTGGTTATCATCGGAGTG, antisense CTCGTTGTTGGAGTTCAGAAGTGG) were assessed. Data were normalised to expression of the housekeeping genes actin and YWHAZ (pre-validated primers purchased from Primerdesign, UK) and analysed for fold changes in gene expression using the comparative CT method with statistical analysis determined using the freely available Relative Expression Software Tool (REST 2009, http://www.qiagen.com).

### HUVEC ELISA for soluble E-selectin

The concentration of soluble E-selectin in cell supernatants derived from HUVEC cells treated with 20 and 200 ng/ml irisin for a period of 4 and 24 h was quantified using soluble E-selectin ELISA (R&D Systems, UK) as per manufacturer’s instructions.

### Statistical analysis

Results are presented as mean ± standard deviation (SD), unless otherwise stated. Comparisons between cohorts and analysis of E-selectin expression data were performed using unpaired t tests. For analysis within the cohort of T2DM individuals, associations between plasma irisin and other circulating factors with anthropometric measures were explored individually using Pearson’s bivariate correlations. Natural log-transformed telomere length was assessed to ensure that associations with T/S ratio always remained non-negative, ensuring that the decline in telomere length with age followed a biologically consistent negative exponential decay model. Subsequently, linear regression was used to investigate whether plasma irisin levels were significantly predictive of plasma soluble E-selectin levels. For all analyses a p value of < 0.05 was considered to be statistically significant.

## Results

### Body anthropometry and biochemical analyses of T2DM patients

Table 1 presents the results of the anthropometric and biochemical analyses in the T2DM study cohort. Overall, the T2DM patients exhibited measurements indicative of central obesity, including an average BMI > 30 kg/m^2^ with high average body fat and abdominal (trunk) fat percentages (34.9 and 34.1%, respectively) and an average visceral fat score above the healthy range (> 13). Biochemical analyses showed an expected elevation of fasting blood glucose (9.9 mmol/l), with concomitantly elevated levels of pro-inflammatory factors, including leptin (1530 ng/ml), C-reactive protein (4.5 μg/ml) and soluble E-selectin (50.9 ng/ml). In the T2DM cohort overnight-fasted, resting, plasma irisin levels were significantly higher than levels previously noted in healthy volunteers (175.4 ± 131 ng/ml vs. 46.7 ± 32.4 ng/ml; p < 0.0001).

**Table 1:** Anthropometric and biochemical measurements in individuals with type 2 diabetes (T2DM)

Anthropometric and biochemical analysis	Type 2 diabetes cohort
Cohort size	79
Men	42
Women	37
Age (years)	56 ± 12
Height (cm)	165 ± 10
Weight (kg)	87 ± 20.5
Body mass index (kg/m^2^)	31.5 ± 5.4
Total fat (%)	34.9 ± 9.3
Total muscle (kg)	53 ± 11.8
Abdominal (trunk) fat (%)	34.1 ± 9.4
Abdominal (trunk) muscle (kg)	29 ± 5.7
Visceral fat score (0–60)	13.2 ± 5.5
Fasting blood glucose (mmol/l)	9.9 ± 3.8
HbA1c (mmol/mol)	70.5 ± 17.6
Fasting insulin (mU/l)	29.1 ± 42.6
HOMA β (%)	67.2 ± 67.9
HOMA-S	74 ± 71
Telomere length (T/S ratio)	1.6 ± 0.2
Irisin (ng/ml)	175.4 ± 131
Leptin (pg/ml)	1529 ± 1372
Thrombomodulin (ng/ml)	6.6 ± 6.3
E-selectin (ng/ml)	50.9 ± 21.4
C-reactive protein (µg/ml)	4.5 ± 3.99

Homeostatic model assessment for beta cell function (HOMA-β) and insulin sensitivity (HOMA-S). Data are presented as mean ± S.D. for normal continuous variables

### Associations of plasma irisin with biochemical and anthropometric factors in type 2 diabetes

**Fig. 1:**
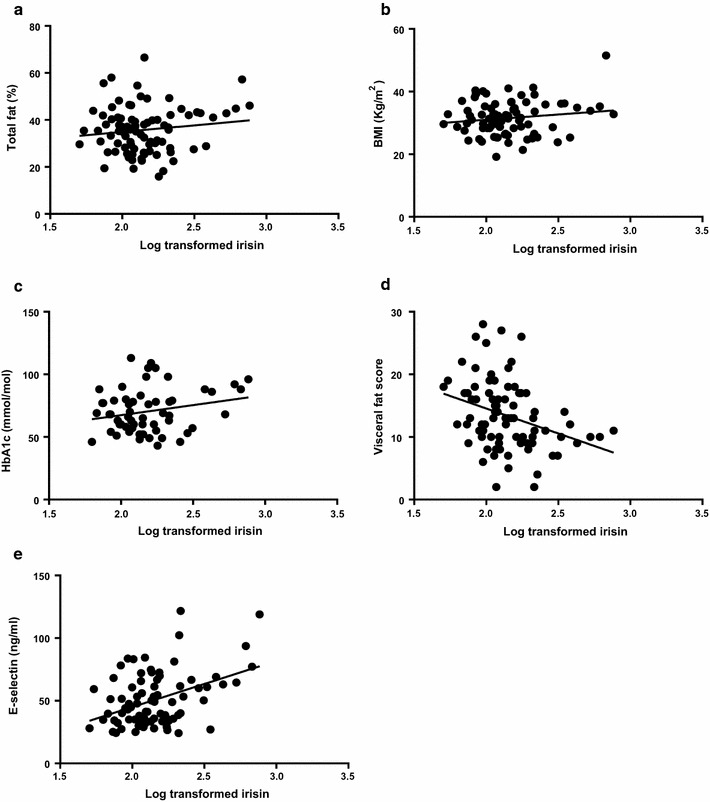
Associations between plasma irisin levels and anthropometric/biochemical measures. In our study cohort of type 2 diabetic patients, Pearson’s correlation tests showed significant positive associations between plasma irisin and body mass index (BMI) (**a** p = 0.04); total fat (%) (**b** p = 0.03); HbA1c (**c** p = 0.032) and E-selectin (**e** p < 0.001); and a significant negative association between plasma irisin levels and the visceral fat score (**d** p = 0.006)

Pearson’s bivariate correlations between plasma irisin and anthropometric and biochemical measures and T/S ratio were assessed and associations are represented in Table 2. BMI (p = 0.04), total fat percentage (p = 0.033), HbA1c (p = 0.032) and E-selectin (p < 0.0001) exhibited significant positive associations with plasma irisin levels in the T2DM cohort. Visceral fat score (p = 0.006) displayed a significant negative association with plasma irisin levels, as did age (p = 0.001) and circulating leptin (p = 0.02). No other factors measured in this study displayed any significant associations with plasma irisin in T2DM subjects, including T/S ratio which, as previously shown, is predicted by plasma irisin in healthy individuals [33]. Figure 1 shows the scatterplots of these associations.

**Table 2:** Results of Pearson’s bivariate correlations analysis

Measurement	β value	p value
Age	− *0.364*	*0.001*
Height	− 0.171	0.13
Weight	0.089	0.43
Body mass index (kg/m^2^)	*0.223*	*0.04*
Total fat (%)	*0.241*	*0.03*
Total muscle (kg)	− 0.134	0.23
Abdominal (trunk) fat (%)	0.062	0.59
Abdominal (trunk) muscle (kg)	− 0.173	0.13
Visceral fat score	− *0.3*	*0.006*
Fasting blood glucose (mmol/l)	− 0.03	0.79
HbA1c (mmol/mol)	*0.28*	*0.03*
Fasting insulin (mU/l)	0.08	0.46
HOMA β (%)	0.01	0.92
HOMA-S	0.05	0.65
Leptin (pg/ml)	− *0.24*	*0.02*
Thrombomodulin (ng/ml)	− 0.16	0.15
E-selectin (ng/ml)	*0.43*	< *0.0001*
C-reactive protein (µg/ml)	0.05	0.62
T/S ratio	− 0.11	0.3

Pearson’s bivariate correlations between anthropometric/biochemical parameters and fasting plasma irisin levels. Homeostatic model assessment for beta cell function (HOMA β) and insulin sensitivity (HOMA-S). Data are represented as positive or negative correlation coefficients with p values < 0.05 indicating statistical significance (in italic text)

### Plasma irisin predicts E-selectin levels in type 2 diabetes

Multivariate regression analysis was used to test the most significant associations with soluble E-selectin. In the T2DM cohort, plasma irisin was significantly predictive for soluble E-selectin, with a β value of 0.382 and an r^2^ value of 24.7 (p = 0.004).

### E-selectin and ICAM-1 gene expression is induced in HUVECs by exposure to high but not low irisin levels

**Fig. 2:**
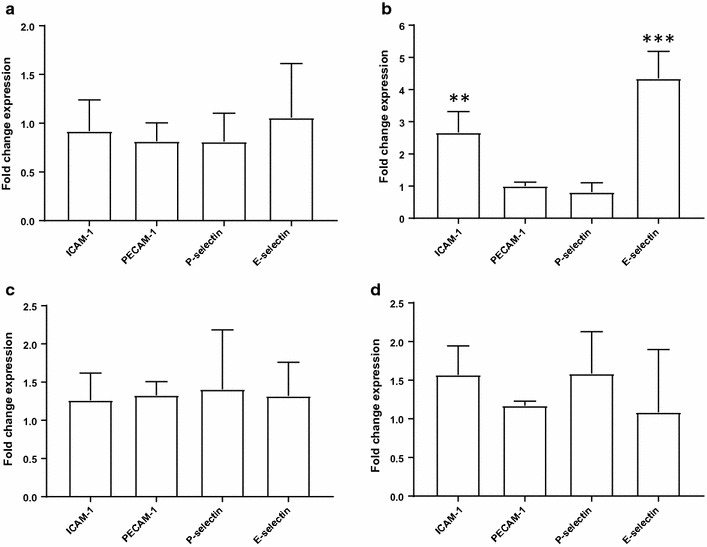
Exposure to recombinant human irisin induces E-selectin and ICAM-1 gene expression in primary HUVECs. Real-time PCR analysis of irisin-treated HUVEC, displaying intracellular adhesion molecule 1 (ICAM1), platelet endothelial cell adhesion molecule (PECAM1), P-selectin and E-selectin mRNA expression levels. **a** Exposure to 20 ng/ml irisin for 4 h caused no changes in gene expression relative to control. **b** Exposure to 200 ng/ml irisin for 4 h induced a fourfold increase in E-selectin and 2.5-fold increase in ICAM-1 mRNA expression compared to control (p = 0.001 and p = 0.014 respectively). **c** Exposure to 20 ng/ml irisin for 24 h caused no significant alteration in expression of genes associated with endothelial dysfunction relative to control and neither did, **d** exposure to 200 ng/ml for 24 h. Values normalised to actin and YWHAZ expression (n = 4). **p<0.01, ***p<0.001 and ****p<0.0001

To investigate the biological mechanism underlying the association between irisin and E-selectin, HUVEC cells were treated for 4 or 24 h with low (20 ng/ml) and high (200 ng/ml) recombinant human irisin. SYBR Green^®^ Real-time PCR analysis of E-selectin and ICAM-1 gene expression showed that exposure to high irisin induced a fourfold and 2.5-fold increase in mRNA expression respectively compared to control (Fig. 2b, p < 0.05). Exposure to low irisin caused no significant difference in E-selectin expression levels (Fig. 2a, p > 0.05). No other markers of endothelial inflammation tested, i.e. P-selectin, and platelet endothelial cell adhesion molecule-1 (PECAM-1), were altered by exposure to irisin. Expression of E-selectin after 24 h of treatment was returned to basal levels (Fig. 2c, d).

### Soluble E-selectin concentration is increased in culture supernatant obtained from HUVECs exposed to high irisin concentrations for 4 h and returns to baseline after 24 h

**Fig. 3:**
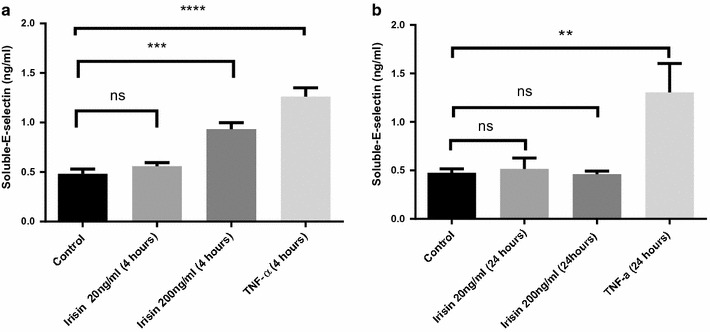
The effect of recombinant human irisin on soluble E-selectin in primary HUVECs. HUVEC cells were treated for **a** 4 and **b** 24 h with recombinant human irisin (20 and 200 ng/ml). TNF-α treatment (10 ng/ml) was used as a positive control (negative control treated only with regular growth media). Supernatant was collected and soluble E-selectin was measured by ELISA. Data was analysed using one way ANOVA with Tukeys multiple comparison test. Data are presented as mean ± SEM; statistical significance was set at p < 0.05. **p<0.01, ***p<0.001 and ****p<0.0001

As measured by ELISA, culture supernatants from HUVECs treated for 4 h with 200 ng/ml recombinant human irisin had 1.8-fold increased soluble E-selectin concentration compared to HUVECs treated with 20 ng/ml of irisin for 4 h (Fig. 3, p < 0.05). Incubation for 24 h with 200 ng/ml irisin resulted in soluble E-selectin concentrations similar to the levels detected in the control group treated with regular growth media. HUVECs treated with TNF-α produced the highest levels of soluble E-selectin (Fig. 3, p ≤ 0.0001) and were used as a positive control.

## Discussion

The current study provides evidence that circulating irisin levels are increased in fasted obese T2DM patients compared with levels which we have previously established for healthy non-diabetic controls [33]. The increased circulating irisin in T2DM showed a significant positive association with plasma soluble E-selectin. The anthropometric analyses in the present study revealed a high BMI with high total and abdominal body fat percentage within the T2DM cohort, whereas the muscle mass of these patients did not differ significantly from values previously reported for a non-diabetic cohort [33]. As irisin is a myokine, this raises the possibility that the increased irisin levels in the T2DM patients may be entering the circulation from a tissue source other than muscle. The strong association between plasma irisin and measures of obesity (including percentage truncal fat), suggest that circulating irisin in this study cohort may be determined by the degree of adiposity. The present results are consistent with previously published data showing correlations between body fat and circulating irisin levels [14, 23, 34].

Although the elevation of circulating irisin in obese T2DM may constitute a compensatory response to decreased energy expenditure, possibly due to lack of exercise or innate defects in metabolism [20, 23] it is also plausible that excess adipose tissue in these patients could provide an extra source of irisin. This is consistent with recent studies in obese subjects showing that plasma irisin decreased markedly in response to a hypocaloric diet and weight loss, together with significant reductions in hyperglycaemia and hyperinsulinaemia [35–37].

It should be also noted that the measurement of irisin in biological samples via immunological methodologies is not without controversy. Indeed, there is some disparity in the reported levels of irisin in serum or plasma depending on the methodology used in detecting it [38]. As such, there is currently no universally accepted ‘reference range’ or normal value for circulating irisin in healthy individuals and some even question its existence [39]. Although it has been suggested that a ‘normal’ circulating concentration of irisin may fall in the narrow range of 3.6–4.6 ng/ml [40], plasma irisin levels similar to those observed in the present study have been independently reported previously [41, 42]. Moreover, the ELISA used in the present study has been previously tested against both Western blotting [43] and mass spectrometry [44].

### Irisin and T2DM

The presently observed plasma irisin concentrations in obese T2DM are interestingly not consistent with previous studies showing lower levels of circulating irisin in T2DM compared to healthy subjects. However, the newly diagnosed T2DM patients in the previous study by Choi et al. were younger, and with a markedly lower BMI compared to the present subjects [45]. Thus, the differences in circulating irisin levels between the studies may be at least partly related to these phenotypic variables. Furthermore, whilst the cohort characteristics in the study by Liu et al. were closer to the present cohort, the higher mean BMI herein may account for the differences in irisin values [46]. The absence of muscle mass and body fat measures in these previous studies makes it difficult to assess the extent of adiposity in their subjects. Additionally, it is possible that differences of ethnicity, diet and parameters identified in the present study may have influenced the irisin levels. The importance of a link between adiposity and circulating irisin cannot be overlooked, particularly if obese individuals are exposed to chronically elevated plasma irisin levels which could potentially confer a state of ‘irisin resistance’. Youn et al, examined serum irisin levels in a cohort of 424 subjects, classifying them according to lower, middle and upper tertiles of skeletal muscle to visceral fat ratio (SVR). Serum irisin was correlated with favourable metabolic phenotypes in those subjects within the upper tertile. However, there were no such correlations in the lower tertile. The authors concluded that circulating irisin is dysfunctionally altered in subjects with lower skeletal muscle mass and higher visceral fat [47] which carries implications for any application of irisin as a potential anti-obesity treatment [5, 12].

Moreover, in the present study a strong positive correlation was noted between HbA1c and plasma irisin levels, suggesting a relationship between circulating irisin levels and glycaemic control. This is supported by previous research that indicated that plasma irisin is associated with elevated blood glucose concentrations [48], whilst evidence also exists that irisin can increase glucose uptake in skeletal muscle cells in vitro in an AMPK-dependent manner [49]. Further work is required therefore to clarify the potential role of irisin in glucose homeostasis and metabolic disease.

### Irisin and soluble E-selectin

In addition to the associations between anthropometric measures and plasma irisin levels, there was a strong positive association between irisin and soluble E-selectin levels. Multiple regression analysis showed that amongst the factors that exhibited a significant association with soluble E-selectin levels (Table 2), soluble E-selectin was the only factor to significantly predict plasma irisin levels. This is an important finding since elevated soluble E-selectin levels are a key marker of endothelial dysfunction in T2DM [50, 51], which accompanies diabetes-related micro- and macro-vascular complications [52–54]. To further explore this hypothesis, we utilized HUVEC cells as a model of the endothelium and observed that exposure to irisin, at concentrations comparable to those in many of the obese T2DM patients of this study, induced a significant increase in E-selectin gene and protein expression (Figs. 2, 3). Since the concentration of soluble E-selectin is directly proportional to its cell surface expression [55], this supports a potential role for irisin in increasing cell surface expression of E-selectin. The precise physiological role of soluble E-selectin is yet to be fully elucidated, although studies have shown that E-selectin can exert a chemotactic signal towards neutrophils, and may trigger T cell migration. Existing literature has reported a positive relationship between soluble E-selectin and pro-inflammatory chemokines, oxidative stress, endothelial dysfunction and incidences of T2DM and CVD [56, 57]. Additionally, Tabak et al. have previously reported a relationship between irisin and low-grade inflammation, a hallmark of metabolic syndrome [58]. Therefore, circulating soluble E-selectin levels are useful as a clinical tool to support a diagnosis of acute inflammatory processes and atherogenic risk [59]. The mechanism(s) of action of irisin in endothelial cell activation remains to be clarified, however the evidence presented here suggests that irisin may provide a novel link between increased adiposity and endothelial dysfunction.

### Conclusion

The benefits of exercise in obesity and diabetes are well known and the prospect of utilising newly discovered hormonal pathways that may mimic these effects is tantalising [60]. The discovery of irisin and its potential role in energy regulation provide a potential step in this direction. Since the initial discovery of irisin by Bostrom et al. there have been several clinical studies investigating associations between circulating irisin and body composition and T2DM [61]. The results so far have been inconsistent, with the exact role of irisin in metabolic disorders yet to be fully elucidated possibly relating to differences in study methodology, which either focus on either skeletal muscle or adipose tissue. The current study considers both tissue types, however the precise mechanisms by which irisin is regulated and released from both muscle and adipose tissue, and also the diverse effects of circulating irisin which impact nutrient homeostasis and endothelial function are yet to be elucidated.

## Abbreviations

BMI: body mass index
BAT: brown adipose tissue
CVD: cardiovascular disease
CRP: C-reactive protein
FFM: fat free mass
FNDC5: fibronectin type III domain-containing
HUVECs: human umbilical vein endothelial cells
IL-1: interleukin-1
ICAM1: intracellular adhesion molecule 1
PECAM-1: platelet endothelial cell adhesion molecule-1
RT-PCR: real-time polymerase chain reaction
FM: segmental fat mass
SVR: skeletal muscle to visceral fat ratio
TL: telomere length
HOMA: the homeostatic model assessment
TNF-α: tumour necrosis factor-alpha
T2DM: type 2 diabetes
UCP1: uncoupling protein-1
WAT: white adipose tissue
